# Development and validation of a tool to assess researchers’ knowledge of human subjects’ rights and their attitudes toward research ethics education in Saudi Arabia

**DOI:** 10.1186/s12910-023-00968-z

**Published:** 2023-11-02

**Authors:** May M. Al-Madaney, Margrit Fässler

**Affiliations:** 1 Institute of Biomedical Ethics and History of Medicine, University of Zurich, Winterthurerstrasse 30, 8006 Zurich, Switzerland; 2 Research Center, King Fahad Medical City, Riyadh Second Health Cluster, P.O. Box. 59046, 11525 Riyadh, Kingdom of Saudi Arabia

**Keywords:** Questionnaire development, Clinical researchers, Research ethics, Knowledge, Attitudes, Saudi Arabia, Validation study

## Abstract

**Background:**

Researchers must adhere to ethical and scientific standards in their research involving human subjects; therefore, their knowledge of human subjects’ rights is essential. A tool to measure the extent of this knowledge is necessary to ensure that studies with participants are conducted ethically and to enhance research integrity. Currently, no validated instrument is available for such an assessment. Therefore, the primary purpose of this study is to develop a reliable and valid instrument to assess researchers’ knowledge of human subjects’ rights in clinical settings, as well as a reliable and valid measure of their attitudes toward clinical research ethics education in Saudi Arabia.

**Methods:**

The current study involves the development of a questionnaire about the rights of human subjects in research and the researchers’ attitudes toward research ethics education. The content was developed based on an extensive review of research ethics guidelines. A panel of experts tested the questionnaire for face validity (*n* = 5) and content validity (*n* = 8). The reliability of the questionnaire was established by a split-half reliability coefficient and item analysis among a sample (*n* = 301) of clinical researchers.

**Results:**

Face validity demonstrated that the questionnaire was quick to complete and easy to answer. The global content validity indices (S-CVIs) were greater than 0.78 for all questionnaire sections; the split-half reliability coefficient was 0.755 for knowledge items; Cronbach’s alpha was 0.77 for researchers’ attitudes, showing good internal consistency. The difficulty index ranged from 12.0% to 98.7% for all knowledge items. Most questions were at an acceptable level of reliability and discrimination criteria. The final version of the questionnaire contained 89 items, distributed as 15 questions on demographic and professional characteristics, 64 questions items on knowledge, and 10 items on attitudes.

**Conclusions:**

The questionnaire is a valid and reliable tool to assess biomedical researchers’ knowledge of human subjects’ rights and their attitudes toward research ethics education. This instrument could help address the gap in researchers’ knowledge of the rights and facilitate the development of educational intervention programs to set appropriate learning objectives.

**Supplementary Information:**

The online version contains supplementary material available at 10.1186/s12910-023-00968-z.

## Background

Clinical research is the gold standard for developing new treatment regimens [1]. Researchers must adhere to ethical and scientific standards when they undertake research involving human subjects [2]. One key objective behind applying these standards is to ensure that any conclusion on treatment effectiveness, mechanisms of disease, normal physiology, and learning and behaviors can be verified through them [3]. Prior to the establishment of such standards, unjustified studies which placed human subjects in harm’s way had taken place without consideration to their rights. This can be seen in past experiments conducted in Nazi Germany during World War II which were the forms of human cruelty, or the Tuskegee Syphilis Study conducted in the United States of America [4].

The principles that form guidelines for ethical research and Good Clinical Practice (GCP) are autonomy, beneficence, non-maleficence, and justice [5]. The well-known Nuremberg Code, issued in 1947, was the beginning of establishing national ethical codes governing medical research [6], followed by the Declaration of Helsinki (1964) which focused on protecting human subjects who participate in medical research [7]. Further, the International Conference on Harmonization Good Clinical Practice (ICH-GCP) (1998) requires that all Institutional Review Boards (IRBs) or Ethics Committees (ECs) are responsible for reviewing research protocols involving human subjects and for ensuring the adequacy of their protection [8].

The Council of Ministers in the Kingdom of Saudi Arabia (KSA) passed a law entitled The Law of Ethics of Research on Living Creatures (the Law) on August 24th, 2010 [9]. Following the passing of the Law, the National Committee of Bioethics (NCBE) issued the Implementing Regulations of the Law of Ethics of Research on Living Creatures (the Implementing Regulations) on December 25th, 2011 [9]. The Saudi system considers Islamic Sharia (Law) in addition to international research ethics guidelines [10]. Therefore, this law serves all Islamic countries in the region with similar values and social structure as those of KSA [11]. Moreover, the Saudi Food and Drug Authority requires clinical trials to follow the ethical principles stated in the ICH-GCP, and World Medical Association—Declaration of Helsinki [12].

Researchers’ knowledge of subjects’ rights is essential for achieving the highest ethical standards [13]. However, despite stringent regulations, studies suggest concerns about the inclusion of informed consent requirements and IRB review may not assure adequate protection for participants [8]. Additionally, studies conducted internationally have indicated insufficient knowledge among faculty members and physicians regarding research ethics [14, 15]. Meanwhile, a multicenter study conducted in KSA and Egypt reported sub-optimal knowledge and attitude related to research ethics among university dental faculties and recommended further studies be carried out to examine the generalizability of their results to other institutions [16].

This indicates a critical need for examining researchers’ knowledge to ensure they are aware of considerations and maintaining human rights throughout any study using validated instruments. Thus, the current study aims to develop a reliable and valid questionnaire on researchers’ knowledge of human subjects’ rights in clinical settings and a reliable and valid measure of their attitudes toward education on research ethics. The current study utilized a sample of medical researchers employed at King Fahad Medical City (KFMC) in KSA. Furthermore, the data resulting from this study may help policymakers develop plans for the effective implementation of ethics committee functions, establish educational intervention programs in clinical research ethics, and subsequently address potential knowledge gaps in these areas.

## Methods

### Study design and setting

The current study involves the development and validation of the questionnaire and then a cross-sectional study conducted at KFMC, one of the main health research centers in Riyadh, Saudi Arabia.

### Study subjects

Participants are KFMC physicians, nurses, pharmacists, technicians, allied health professionals, and medical researchers (principal investigators, co-investigators, and research coordinators) who have conducted at least one research study, were listed in the KFMC IRB database from 2007 to 2021, and were willing to participate in the study. Furthermore, health care workers who have participated in any of the conducted research were polled to gain insight into the ethical conduct of the research.

### Data collection and management

An invitation letter was sent through email to the 550 active researchers in the IRB records at KFMC who met the criteria to participate in the study. Attached to the invitation letter was the Google Form link to the questionnaire that contained the cover letter describing the study and requiring the researchers’ consent for voluntary participation. When our sample size of 301 participants signed up through the Google Form link, no more could sign up. The questionnaire was in the English language, which is the main working language for the staff (researchers) at KFMC. Only the research team had access to the data, and anonymity and confidentiality were always maintained.

### Questionnaire development

**Fig. 1:**
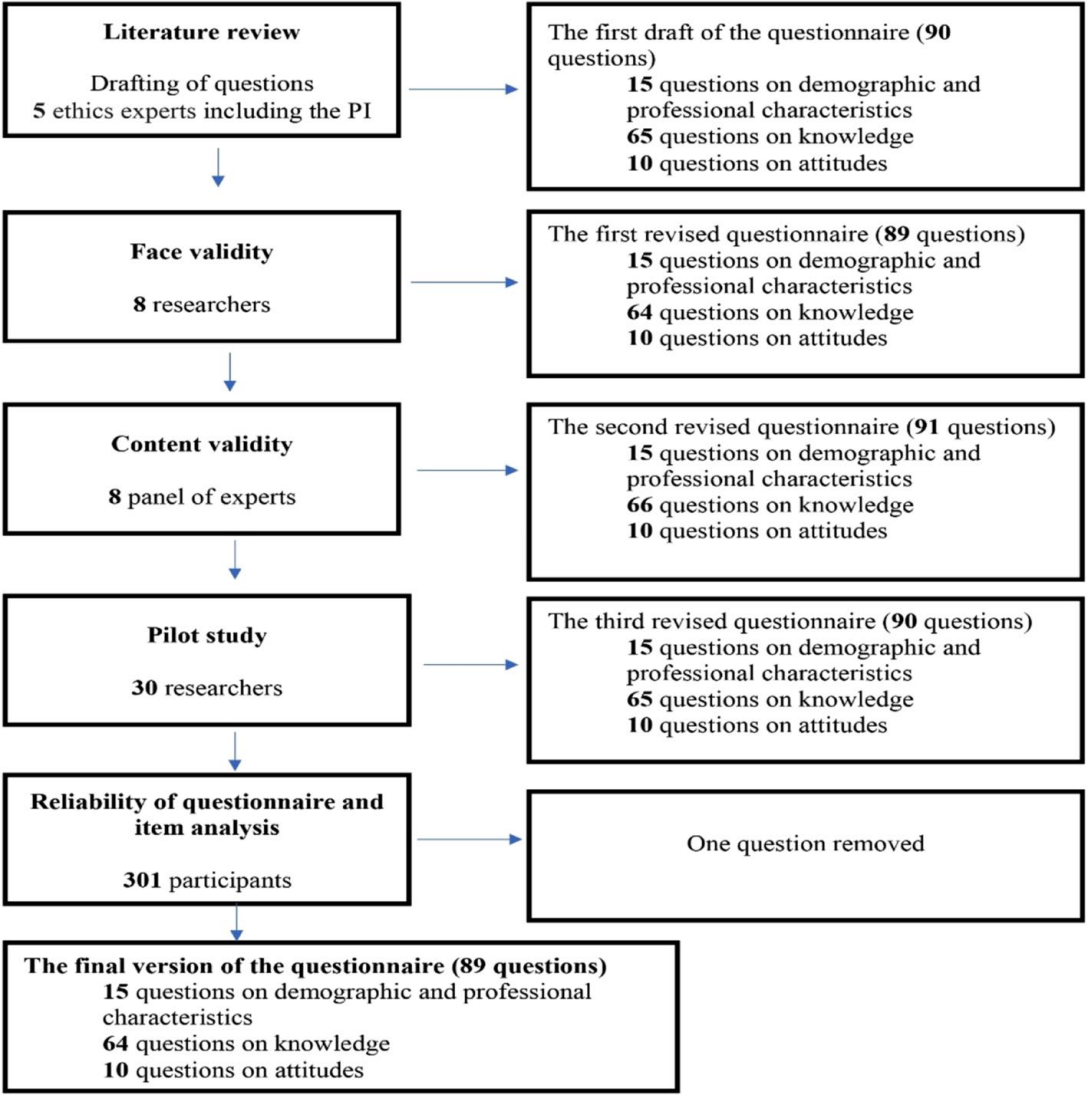
Flow chart outlining the development and validity of the questionnaire

Five important steps were taken in the development of the questionnaire used to evaluate and assess researchers’ knowledge of the rights of human subjects in research. We searched the literature extensively for a questionnaire. Figure 1 presents a flow chart outlining the development and validity of the questionnaire.

The questionnaire was developed from a previous study [16] and obtained from issues addressed in the ethical guidelines in the ICH-GCP and Code of Federal Regulations [17, 18]. Five ethics experts, including the principal investigator, belonging to different nationalities (USA, Switzerland, Germany, and Saudi Arabia) were involved in developing the first draft of the questionnaire.

The questionnaire was divided into three sections. The first section covered respondents’ demographic information and professional characteristics, including their age, gender, nationality, education level, whether they were medical education graduates, occupation, years of research experience, and the number of research publications in medical journals. The second section explored respondents’ knowledge of subjects’ rights in clinical research. The third section explored respondents’ attitudes toward education on research ethics. To the questions in the first section a single response, multiple responses, or “yes,” “no,” or “not sure” responses were required; answers in the second section were assessed with “correct,” “not correct,” or “I don’t know” choices. Finally, answers in the third section were assessed on a 5-point Likert scale (strongly agree, agree, neutral, disagree, and strongly disagree).

### Face validity

Face validity is used to assess readability, feasibility, and consistency of the style and formatting, and clarity of the language used in the questionnaire’s appearance [19, 20]. Eight researchers reviewed the first draft of the questionnaire (see Additional file 1).

To determine the face validity of the questionnaire, we created an evaluation form that helps participants assess various items. It also helped us identify areas for improvement and potential additions to the next version. The questions were evaluated for clarity, style, ease of understanding, and layout.

### Content validity

The questionnaire, after face validation, was sent to a panel of eight content experts in the field of research ethics to review the instrument for content validity. These experts included professors and persons with PhDs in the field of bioethics with vast experience in human research and belonged to different nationalities (USA, Switzerland, Germany, Saudi Arabia). They reviewed the questionnaire for readability, clarity, and comprehensiveness and reached a level of agreement on which questions should be retained in the final questionnaire.

For content validity, the panel reviewed the relevance of each question on a 4-point Likert scale: 1 = not relevant, 2 = somewhat relevant, 3 = relevant, and 4 = very relevant. Then for each question, the number of experts giving a 3 or 4 score is counted (3,4 – relevant; 1,2 – non-relevant). The content validity index (CVI) was calculated by averaging the cumulative level of agreement among the experts [21, 22]. To assess content validity, we used the content validity of individuals (I-CVI) and the overall scale (S-CVI). The scale-level content validity indices (S-CVI) were calculated from the item-level content validity index (I-CVI) [20, 21, 23]. Previous research suggested that an I-CVI of ≥ 0.78 obtained from 6 to10 raters would be acceptable for inclusion in the questionnaire [23].

### Pilot study and questionnaire revision

An initial form was pilot tested on a convenience sample of 30 consenting doctors, pharmacists, nurses, and other researchers at KFMC. The investigator asked each respondent to keep track of the amount of time it took to complete the questionnaire which took approximately 20–25 min to complete, and they provided feedback on any misleading or confusing question items.

Further, the investigator wanted to ensure that the questions were easy for all respondents to understand. The participants’ suggestions included reformulating and rewording some items and removing potentially repetitive ones. Furthermore, five experts reviewed the pilot study results— two from the Clinical Trials Center at University of Zurich and three having expertise in research ethics. All reported feedback was sent to the principal investigator for necessary actions. Questions were removed or modified based on the suggestions.

### Questionnaire reliability

Because the investigator could not repeat the test, the current study evaluated the internal consistency of knowledge items in the questionnaire using the split-half coefficient. When re-testing is not possible, the split-half coefficient is an alternative technique for the assessment of questionnaire reliability through the division of questions into two halves (e.g., odd versus even). The values of the split-half coefficient range from 0.0 to 1.0—higher values indicate high reliability of the test [24]. Conversely, Cronbach’s alpha was reported to assess the reliability of the section that contains researchers’ attitudes toward education about research ethics because it is measured using a 5-point Likert scale [25].

To do so, we selected a random sample from the population of registered researchers in the IRB records at KFMC, which had a total population size of 550 active researchers. Therefore, to establish a power of 80% at the 99% confidence interval with a 5% margin of error, the required sample size was 301 participants. Therefore, we conducted a reliability analysis using a sample of 301 researchers drawn randomly from the IRB records at KFMC. The questionnaire was distributed to participants using a Google Form link, along with a cover letter explaining the purpose of the study and the confidentiality of their personal data.

### Item analysis

The item analysis is used to assess the effectiveness of each question in a certain test. It can be performed using item-difficulty and item-discrimination indices [26, 27]. The difficulty index measures the percentage of participants who correctly answered each item. The values in the item-difficulty index ranged from 0.0 to 1.0 (0.0 to 100%) — its higher values indicate the greater difficulty of the question and lower values indicate the lesser difficulty. The ideal item-difficulty index is 85% for dichotomous questions (correct/ incorrect) and 77% for three multiple choice questions [26]. The questions that showed item difficulty ≤ 30.0% indicated great difficulty (difficult); between 30 and 80% showed medium or moderate difficulty; and ≥ 80% showed less difficulty (easy) [26].

Meanwhile, the item-discrimination index for each item distinguishes between participants who do well and those who do poorly in a test [27]. It can be measured by dividing the sample into high- and low- skill groups based on the total test score, and then the discrimination index can be calculated by subtracting the percentage of those who correctly answered the item in the low-skill group (i.e., bottom 25%) from the percentage of those who correctly answered the item in the high-skill group (i.e., top 25%). Its values range from -1.0 to 1.0 — higher positive values indicate that the question discriminates between the two groups, while lower values indicate poorer discrimination. Negative values near -1.0 indicate that participants in the low-skill group correctly answered the question; however, they incorrectly did it compared to those in the high-skill group [28]. Another way to calculate item discrimination is the point-biserial correlation, which measures the correlation between each question and the total test score (i.e., item-to-total correlations). Table 1 shows the guidelines to determine whether the question should be rejected or improved [27]. On the other hand, the question that has a point-biserial correlation < 0.20 is to be removed or revised [28]. The data collected during reliability analysis was also used in the item analysis.

**Table 1:** Discrimination guidelines to determine whether the question should be rejected or improved

		**Item difficulty**	
Item discrimination (*d*)	High	Medium or moderate	Low (easy)
*d* $\le$ 0.0	Improve or reject	Improve or reject	Improve or reject
0.0 < *d* $\le$ 0.30	Accept	Improve or reject	Accept
*d* > 0.30	Accept	Accept	Accept

### The final version of questionnaire

Based on the validity and reliability testing of the previous steps specified above, we have produced the final questionnaire.

### Ethical considerations

This study was conducted following the ethical requirements of KFMC. Ethical approval was obtained from the IRB at KFMC (IRB log No. 19–240). All methods were performed in accordance with the relevant guidelines and regulations or declaration of Helsinki. The potential participants’ contact details were obtained from the IRB at KFMC subsequent to ethical approval. The participants were informed that privacy and anonymity would be maintained.

### Statistical analysis

Content validity was assessed by calculating I-CVI and S-CVI for each part in the questionnaire. The split-half reliability coefficient was calculated to assess the internal consistency reliability of the items used to assess researchers’ knowledge. Cronbach’s alpha was calculated to assess the internal consistency and reliability of each item used to assess researchers’ attitudes toward research ethics and education with the other items. Item analysis was conducted using the difficulty index and the discrimination index (discrimination and point-biserial correlations). Descriptive statistics of the characteristics related to demographic and research background were presented in terms of mean (standard deviation; SD), median (interquartile range; IQR), or counts and percentages as appropriate. We also used independent samples t-tests and one-way ANOVA with Tukey–Kramer post hoc analysis to compare average total knowledge scores by demographics and between trained and untrained participants. Statistical significance was sought at values < 0.05. All analyses were done using a standard software package (Stata, version 15.0; StataCorp).

## Results

### Questionnaire design

The first draft of the questionnaire about researchers’ knowledge on human subjects’ rights and attitudes toward research ethics education consisted of three parts and a total of 90 questions (see Additional file 1).

- Part I- The demographic and professional characteristics, which contained 15 questions.
- Part II- The knowledge section that contained six sections and a total of 65 questions is detailed below:
  - Section 1: The basic and additional elements of informed consent: 17 Questions
  - Section 2: Institutional Review Board (IRB) or Research Ethics Committee (REC): 10 Questions
  - Section 3: Safety Reporting Issues in Clinical Research: 6 Questions
  - Section 4: Researchers’ responsibilities in clinical research: 19 Questions
  - Section 5: Technical Aspects of the Informed Consent Process: 10 Questions
  - Section 6: Clinical research scenario on confidentiality: 3 Questions
- Part III- The researchers’ attitude toward research ethics education, which contained 10 questions.

### Face validity

All respondents reviewed each of the 65 questions on researchers’ knowledge and the 10 questions on researchers’ attitudes. The respondents indicated that they understood the questions. Eight respondents found these questions easy to answer and four of these eight respondents suggested that the appearance and layout would be acceptable to the target audience. Most respondents suggested splitting question 11, in Part II, Section 1 into two questions to ensure clarity, and redrafting question 10 in Part II, Section 2. Most reviewers suggested removing question 18 and redrafting question 19 in Part II, Section 4. They suggested removing question 7 in Part II, Section 5, because of its ambiguity and adding a new question to ensure clarity. They suggested removing question 2 and redrafting question 3 in Part II, Section 6. At the end of face validity, the questionnaire had a total of 74 questions (consisting of 64 questions on knowledge and 10 questions on researchers’ attitudes toward research ethics education). After this, the questionnaire was sent for content validity (see Additional file 2).

### Content validity

The panel of experts reviewed 74 questions (64 questions on the knowledge of human subjects’ rights and 10 questions on researchers’ attitudes toward research ethics education). For questions with a factual statement in the knowledge section, the panel suggested changing the answer options from “yes,” “no,” and “I don’t know” to “correct,” “not correct,” and “I don’t know.”

The content validity process resulted in the addition, revision, and redrafting of some questions according to the panel’s opinions. As a result of the panel’s input, the revised questionnaire contained 76 questions and 74 response items. At the end of content validity, the questionnaire had a total of 76 questions, consisting of 66 questions on knowledge and 10 questions on researchers’ attitudes toward research ethics education (see Additional file 3). Tables 2, 3, 4, 5, 6 and 7 show the item-level content validity index I–CVI for the six sections in Part II of the questionnaire about knowledge (Sections 1–6) as 0.99, 0.99, 1.00, 0.97, 0.95, and 1.00, respectively. For attitudes (part III of the questionnaire), the I-CVI is 0.95 (Table 8).

**Table 2:** Fictitious rating on an 18-item scale by eight experts (Part II, Section 1)

**Item**	**Expert 1**	**Expert 2**	**Expert 3**	**Expert 4**	**Expert 5**	**Expert 6**	**Expert 7**	**Expert 8**	**Number in Agreement**	**Item CVI**
1	x	x	x	x	x	x	x	x	8	1.00
2	x	x	x	x	x	x	_	x	7	0.88
3	x	x	x	x	x	x	x	x	8	1.00
4	x	x	x	x	x	x	x	x	8	1.00
5	x	x	x	x	x	x	x	x	8	1.00
6	x	x	x	x	x	x	x	x	8	1.00
7	x	x	x	x	x	x	x	x	8	1.00
8	x	x	x	x	x	x	x	x	8	1.00
9	x	x	x	x	x	x	x	x	8	1.00
10	x	x	x	x	x	x	x	x	8	1.00
11	x	x	x	x	x	x	x	x	8	1.00
12	x	x	x	x	x	x	x	x	8	1.00
13	x	x	x	x	x	x	x	x	8	1.00
14	x	x	x	x	x	x	x	x	8	1.00
15	x	x	x	x	x	x	x	x	8	1.00
16	x	x	x	x	x	x	x	x	8	1.00
17	x	x	x	x	x	x	x	x	8	1.00
18	x	x	x	_	x	x	x	x	7	0.88
									Mean I-CVI =	0.99
Proportion Relevant:									S-CVI/UA =	0.89
	1.00	1.00	1.00	0.90	1.00	1.00	0.90	1.00	Mean expert proportion =	0.98

*I-CVI* item-level content validity index, *S-CVI/UA* scale-level content validity index, universal agreement calculation method

**Table 3:** Fictitious rating on a 10-item scale by eight experts (Part II, Section 2)

**Item**	**Expert 1**	**Expert 2**	**Expert 3**	**Expert 4**	**Expert 5**	**Expert 6**	**Expert 7**	**Expert 8**	**Number in Agreement**	**Item CVI**
1	x	x	x	x	x	x	x	x	8	1.00
2	x	x	x	x	x	x	x	x	8	1.00
3	x	x	x	_	x	x	x	x	7	0.88
4	x	x	x	x	x	x	x	x	8	1.00
5	x	x	x	x	x	x	x	x	8	1.00
6	x	x	x	x	x	x	x	x	8	1.00
7	x	x	x	x	x	x	x	x	8	1.00
8	x	x	x	x	x	x	x	x	8	1.00
9	x	x	x	x	x	x	x	x	8	1.00
10	x	x	x	x	x	x	x	x	8	1.00
									Mean I-CVI =	0.99
Proportion Relevant:									S-CVI/UA =	0.90
	1.00	1.00	1.00	0.90	1.00	1.00	1.00	1.00	Mean expert proportion =	0.99

**Table 4:** Fictitious rating on a 6-item scale by eight experts (Part II, Section 3)

**Item**	**Expert 1**	**Expert 2**	**Expert 3**	**Expert 4**	**Expert 5**	**Expert 6**	**Expert 7**	**Expert 8**	**Number in Agreement**	**Item CVI**
1	x	x	x	x	x	x	x	x	8	1.00
2	x	x	x	x	x	x	x	x	8	1.00
3	x	x	x	x	x	x	x	x	8	1.00
4	x	x	x	x	x	x	x	x	8	1.00
5	x	x	x	x	x	x	x	x	8	1.00
6	x	x	x	x	x	x	x	x	8	1.00
									Mean I-CVI =	1.00
Proportion Relevant:									S-CVI/UA =	1.00
	1.00	1.00	1.00	1.00	1.00	1.00	1.00	1.00	Mean expert proportion =	1.00

**Table 5:** Fictitious rating on an 18-item scale by eight experts (Part II, Section 4)

**Item**	**Expert 1**	**Expert 2**	**Expert 3**	**Expert 4**	**Expert 5**	**Expert 6**	**Expert 7**	**Expert 8**	**Number in Agreement**	**Item CVI**
1	x	x	x	x	x	x	x	x	8	1.00
2	x	x	x	x	x	x	x	x	8	1.00
3	x	x	x	x	x	x	x	x	8	1.00
4	x	x	x	x	x	x	x	x	8	1.00
5	x	x	x	_	x	x	x	x	7	0.88
6	x	x	x	x	x	x	x	x	8	1.00
7	x	x	x	x	x	x	x	x	8	1.00
8	x	x	x	_	x	x	x	x	7	0.88
9	x	x	x	x	x	x	x	x	8	1.00
10	x	x	x	x	x	x	x	x	8	1.00
11	x	x	x	x	x	x	x	x	8	1.00
12	x	x	x	x	x	x	x	x	8	1.00
13	x	x	x	_	x	x	x	x	7	0.88
14	x	x	x	x	x	x	x	x	8	1.00
15	x	x	x	x	x	x	x	x	8	1.00
16	x	x	x	x	x	x	_	x	7	0.88
17	x	x	x	x	x	x	x	x	8	1.00
18	x	x	x	x	x	x	x	x	8	1.00
									Mean I-CVI =	0.97
Proportion Relevant:									S-CVI/UA =	0.78
	1.00	1.00	1.00	0.80	1.00	1.00	0.90	1.00	Mean expert proportion =	0.96

**Table 6:** Fictitious rating on a 10-item scale by eight experts (Part II, Section 5)

**Item**	**Expert 1**	**Expert 2**	**Expert 3**	**Expert 4**	**Expert 5**	**Expert 6**	**Expert 7**	**Expert 8**	**Number in Agreement**	**Item CVI**
1	x	x	x	x	x	x	x	x	8	1.00
2	x	x	x	x	x	x	x	x	7	1.00
3	x	x	x	_	x	x	x	_	6	0.75
4	x	x	x	x	x	x	x	x	8	1.00
5	x	x	x	x	x	x	x	x	8	1.00
6	x	x	x	x	x	x	x	x	8	1.00
7	x	x	x	x	x	x	x	x	8	1.00
8	x	x	x	x	x	x	x	_	7	0.88
9	x	x	x	x	x	x	x	_	7	0.88
10	x	x	x	x	x	x	x	x	8	1.00
									Mean I-CVI =	0.95
Proportion Relevant:									S-CVI/UA =	0.70
	1.00	1.00	1.00	0.80	1.00	1.00	1.00	0.70	Mean expert proportion =	0.94

**Table 7:** Fictitious rating on a 2-item scale by eight experts (Part II, Sect. 6)

**Item**	**Expert 1**	**Expert 2**	**Expert 3**	**Expert 4**	**Expert 5**	**Expert 6**	**Expert 7**	**Expert 8**	**Number in Agreement**	**Item CVI**
1	x	x	x	x	x	x	x	x	8	1.00
2	x	x	x	x	x	x	x	x	8	1.00
									Mean I-CVI =	1.00
Proportion Relevant:									S-CVI/UA =	1.00
	1.00	1.00	1.00	1.00	1.00	1.00	1.00	1.00	Mean expert proportion =	1.00

**Table 8:** Fictitious rating on a 10-item scale by eight experts (Part II, Section 5)

**Item**	**Expert 1**	**Expert 2**	**Expert 3**	**Expert 4**	**Expert 5**	**Expert 6**	**Expert 7**	**Expert 8**	**Number in Agreement**	**Item CVI**
1	x	x	x	x	x	x	x	x	8	1.00
2	x	x	x	x	x	x	x	x	8	1.00
3	x	x	x	x	x	x	x	x	8	1.00
4	x	x	x	x	x	x	x	x	8	1.00
5	x	x	x	x	x	x	x	x	8	1.00
6	x	x	x	x	x	x	x	x	8	1.00
7	x	x	x	x	x	x	x	x	8	1.00
8	x	_	_	x	x	x	x	x	6	0.75
9	x	_	_	x	x	x	x	x	6	0.75
10	x	x	x	x	x	x	x	x	8	1.00
									Mean I-CVI =	0.95
Proportion Relevant:									S-CVI/UA =	0.80
	1.00	0.80	0.80	1.00	1.00	1.00	1.00	1.00	Mean expert proportion =	0.95

### Pilot study and questionnaire revision

A total of 30 doctors, nurses, pharmacists, and researchers participated in the pilot study and were not a part of the 301 researchers who participated in the questionnaire reliability assessment. The questionnaire consisting of 76 questions (66 questions on knowledge and 10 on their attitudes toward research ethics education) was distributed to the participants. Though the participants understood the questions, they proposed to reword and delete some questions. Finally, the experts suggested adding some questions to the knowledge part related to the IRB and safety reporting issues in clinical research.

Most respondents suggested to redraft question 9 in part II, Section 1 to ensure clarity, to delete question 4 in part II, Section 2, and to delete questions 8 and 11 because they were negatively worded questions causing confusion and suggested adding two new questions to ensure clarity. The respondents suggested modifying question No. 4 in Part II, Section 3, to ensure clarity and adding a new question. The majority of reviewers suggested that question 18 in Part II, Section 4 be removed, and question 10 be redrafted. They proposed modifying and redrafting questions 3, 5, and 9 in Part II, Section 5. At the end of the pilot study, the questionnaire consisted of 75 questions—65 questions on researchers’ knowledge and 10 on their attitudes toward research ethics education. These questions were used in the item analysis questionnaire (see Additional file 4).

### Questionnaire reliability

A total of 301 participants completed the questionnaire. Of them, there were 78 (25.9%) consultants; 62 (20.6%) assistant consultants; 21 (7.0%) fellows; 18 (6.0%) residents; 31 (10.3%) pharmacists; 5 (1.7%) faculties; 51 (16.9%) nurses; 35 (11.6%) from other occupations. Moreover, 153 (50.8%) were males; 103 (34.2%) were aged between 31 and 40 years; 188 (62.5%) were Saudis; 144 (47.8%) had a master’s degree; 89 (29.6%) graduated from Saudi Arabia. Among the 301 participants, 235 (78.1%) had prior training in the ethics of protecting research subjects’ rights; 239 (79.4%) participated in research as principal investigators; 283 (94.0%) participated in research as co-investigators; 210 (69.8%) had knowledge of ethical guidelines. The median years of experience was 10 (IQR: 16–6) years, and the median number of publications was 14 (IQR: 27–6) articles. The demographic information and research background of the participants are shown in Table 9.

**Table 9:** Descriptive statistics of the study participants’ demographic and professional characteristics

**Characteristics**	**N**	**%**
**Gender**		
Male	153	50.8
Female	148	49.2
**Age in years**		
$\le$ 30	46	15.3
31–40	103	34.2
41–50	100	33.2
> 50	52	17.3
**Nationality**		
Saudi	188	62.5
Non-Saudi	113	37.5
**Level of education**		
Bachelor	65	21.6
Masters	144	47.8
Doctorate or Ph.D	53	17.6
Subspecialty or Fellowship	39	13.0
**Medical education graduates**		
Saudi Arabia	89	29.6
Arab countries	43	14.3
Europe	60	19.9
North America	72	23.9
Other	37	12.3
**Occupation**		
Consultant	78	25.9
Assistant consultant	62	20.6
Fellow	21	7.0
Resident	18	6.0
Pharmacist	31	10.3
Faculty	5	1.7
Nurse	51	16.9
Other	35	11.6
**Training in Human Research Subjects’ Protection ethics, yes**	235	78.1
*Good Clinical Practice (GCP) guidelines*	*208*	*88.5*
*National Institutes of Health (NIH) Research Ethics*	*151*	*64.2*
*Collaborative Institutional Training Initiative (CITI) Program*	*22*	*9.4*
*The Saudi National Committee of Bioethics (NCBE) certification*	*91*	*38.7*
*The National Institute on Drug Abuse (NIDA) Clinical Trials Network*	*24*	*10.2*
**Principal investigator**		
Yes	239	79.4
No	62	20.6
**Co-investigator**		
Yes	283	94.0
No	18	6.0
**How well do you know the “World Medical Association Declaration of Helsinki Ethical Principles for Medical Research Involving Human Subjects”?**		
Excellent knowledge	74	24.6
Very good knowledge	93	30.9
Average knowledge	86	28.6
Very little knowledge	32	10.6
I don’t know what the Declaration of Helsinki is	16	5.3
**How well do you know the regulations of the law of ethics of research on living creatures issued by “The Saudi National Committee of Bioethics (NCBE)”?**		
Excellent knowledge	59	19.6
Very good knowledge	91	30.2
Average knowledge	65	21.6
Very little knowledge	52	17.3
I am not aware of the Saudi National Committee of Bioethics	34	11.3
**Ethical guideline knowledge**		
Yes	210	69.8
No	91	30.2
**Research experience, median (IQR**^**a**^**)**	10	(16 – 6)
**Number of publications, median (IQR)**	14	(27 – 6)
**Total knowledge score**		
Mean (SD)	52.4	(4.4)
Median (IQR)	52.0	(55.0 – 48.0)
**Total opinion score**	52.4	
Mean (SD)	42.5	(4.4)
Median (IQR)	43.0	(45.5 – 40.0)

^a^*IQR* Interquartile range

The coefficient of split-half reliability for the resulting 65 knowledge questions was 0.755 for the items in the total questionnaire, demonstrating a good internal consistency and reliability of the questionnaire with an average total score of 52.4 ± 9.1 out of the maximum 65 points (80.6%). The Cronbach’s alpha for the researchers’ attitudes toward education of research ethics was 0.77 (95% CI: 0.73 to 0.81), demonstrating good reliability with an average total score of 42.5 ± 4.4 out of the maximum 50 points (85.0%), indicating a good perception of research ethics education. The results in Table 10 indicate most participants’ positive opinions on education about research subjects’ rights. Notably, the participants preferred face-to-face teaching methods in comparison to distance learning. Moreover, all items significantly contributed to acceptable internal consistency. However, if item 8.5 was deleted, the internal consistency reliability of the remaining 9 items would have led to a higher internal consistency reliability (i.e., 0.848), but we retained this item to discriminate between face-to-face and distance learning and to understand the extent to which the participants supported distance learning.

**Table 10:** The item deleted mean and Cronbach’s if item deleted for attitudes section

**Items**	**Mean (SD)**	**Item result**^**a**^	**Cronbach’s Alpha if Item Deleted**
8.1 Research ethics should be taught as a mandatory undergraduate module	4.53 (0.64)	Strongly agree	0.749
8.2 Research ethics should be taught as a mandatory postgraduate module	4.58 (0.65)	Strongly agree	0.718
8.3 All investigators should have some training in research ethics	4.62 (0.66)	Strongly agree	0.727
8.4 I prefer face-to-face as the most effective research ethics teaching methods	4.18 (0.89)	Agree	0.764
8.5 I prefer distance-learning as the most effective research ethics teaching methods	2.95 (1.00)	Neutral	0.848
8.6 I prefer hands-on and case scenarios as a teaching method	4.37 (0.81)	Strongly agree	0.727
8.7 I think is useful to have a research ethics post education exam to assess my knowledge	4.02 (0.81)	Agree	0.731
8.8 I would like to read course materials in advance of the ethics training course	4.05 (0.74)	Agree	0.733
8.9 Research ethics education should be mandatory for health care professionals	4.45 (0.83)	Strongly agree	0.733
8.10 The IRB members should be educated in research ethics	4.76 (0.53)	Strongly agree	0.737

^a^If the item mean ranges from 1.00 to 1.79: strongly disagree; from 1.80 to 2.59: disagree; from 2.60 to 3.39: neutral; from 3.40 to 4.19: agree; from 4.20 to 5.0: strongly agree

Therefore, all 65 knowledge questions met the criteria of reliability and were at acceptable levels of reliability like the 10 items on researchers’ attitudes toward education on research ethics. Thus, the pre-final questionnaire consisted of 75 items—65 knowledge items and 10 items on researchers’ attitudes toward education on research ethics.

### Item analysis

Table 11 shows the item-difficulty and item-discrimination analysis of the knowledge items in the questionnaire. The results of the item analysis showed that most questions were at appropriate levels of difficulty and discrimination as per the guidelines in (Table 1). However, we found that five questions had unacceptable item difficulty and item discrimination (i.e., Q 2.2, Q 2.10, Q 2.15, Q 5.11, and Q 6.3). Questions 2.2 and 2.10 were retained because they measured the basic aspects of informed consent and received an I-CVI of 0.88 and 1.00 in content validity analysis, respectively. Question 2.15 was retained in the questionnaire because it is considered one of the additional elements of the informed consent that was taken from the Code of Federal Regulations (CFR) [18]. Question 5.11 was maintained because it measured knowledge of researchers’ responsibilities in clinical research; also, this question performed well in content validity and had an I-CVI of 1.00. These questions were important to ensure that respondents did not skim-read the question and randomly provide the answer (e.g., superficial answers) and reduce acquiescence and extreme response biases (i.e., Q2.2, Q2.10, and Q5.11). Question 6.3 was discarded from the questionnaire as it also had a low I-CVI (i.e., 75.0%). Therefore, at the end of item analysis, the final version of the questionnaire consisted of 74 question items, of which 64 were about researchers’ knowledge about human subjects’ rights and 10 were about researchers’ attitudes toward research ethics education (see Additional file 5).

**Table 11:** Item difficulty and discrimination

**Items**	**Difficulty**	**Discrimination**	**R**
**Q 2.1**	0.893	0.09	0.05
**Q 2.2**	0.701	0.16^*^	*0.17*
**Q 2.3**	0.897	0.13	0.23
**Q 2.4**	0.907	0.12	0.14
**Q 2.5**	0.907	0.16	0.17
**Q 2.6**	0.953	0.14	0.31
**Q 2.7**	0.890	0.06	0.08
**Q 2.8**	0.903	0.12	0.16
**Q 2.9**	0.910	0.20	0.34
**Q 2.10**	0.571	0.09^*^	*0.08*
**Q 2.11**	0.940	0.17	0.25
**Q 2.12**	0.887	0.19	0.25
**Q 2.13**	0.983	0.04	0.20
**Q 2.14**	0.914	0.15	0.23
**Q 2.15**	0.761	0.26^*^	*0.15*
**Q 2.16**	0.811	0.18	0.13
**Q 2.17**	0.714	0.32	0.18
**Q 2.18**	0.804	0.30	0.20
**Q 2.19**	0.565	0.30	0.20
**Q 3.1**	0.987	0.03	0.23
**Q 3.2**	0.927	0.14	0.27
**Q 3.3**	0.930	0.11	0.24
**Q 3.4**	0.904	0.17	0.20
**Q 3.5**	0.940	0.10	0.16
**Q 3.6**	0.977	0.03	0.18
**Q 3.7**	0.595	0.33	0.30
**Q 3.8**	0.854	0.07	0.13
**Q 3.9**	0.940	0.09	0.12
**Q 3.10**	0.216	0.28	0.24
**Q 4.1**	0.900	0.08	0.12
**Q 4.2**	0.860	0.23	0.25
**Q 4.3**	0.887	0.21	0.22
**Q 4.4**	0.120	0.06	0.03
**Q 4.5**	0.694	0.28	0.22
**Q 4.6**	0.924	0.13	0.19
**Q 4.7**	0.203	0.10	0.13
**Q 5.1**	0.960	0.09	0.35
**Q 5.2**	0.877	0.16	0.25
**Q 5.3**	0.957	0.09	0.22
**Q 5.4**	0.957	0.10	0.25
**Q 5.5**	0.894	0.24	0.32
**Q 5.6**	0.914	0.15	0.16
**Q 5.7**	0.937	0.06	0.14
**Q 5.8**	0.542	0.28	0.23
**Q 5.9**	0.900	0.21	0.33
**Q 5.10**	0.897	0.09	0.16
**Q 5.11**	0.601	0.25^*^	0.16
**Q 5.12**	0.804	0.05	0.08
**Q 5.13**	0.654	0.41	0.31
**Q 5.14**	0.864	0.22	0.30
**Q 5.15**	0.894	0.08	0.15
**Q 5.16**	0.691	0.38	0.35
**Q 5.17**	0.870	0.18	0.26
**Q 6.1**	0.937	0.10	0.11
**Q 6.2**	0.920	0.11	0.20
**Q 6.3**	0.316	0.16^*^	0.08
**Q 6.4**	0.877	0.22	0.30
**Q 6.5**	0.625	0.41	0.33
**Q 6.6**	0.917	0.12	0.23
**Q 6.7**	0.973	0.07	0.26
**Q 6.8**	0.535	0.35	0.25
**Q 6.9**	0.728	0.25	0.21
**Q 6.10**	0.854	0.13	0.18
**Q 7.1**	0.814	0.35	0.33
**Q 7.2**	0.874	0.26	0.28

R: point-biserial correlation (item-to-total correlation)

^*^Indicates a not-significant *p*-value and the fact that the question was rejected or improved by revision

**Fig. 2:**
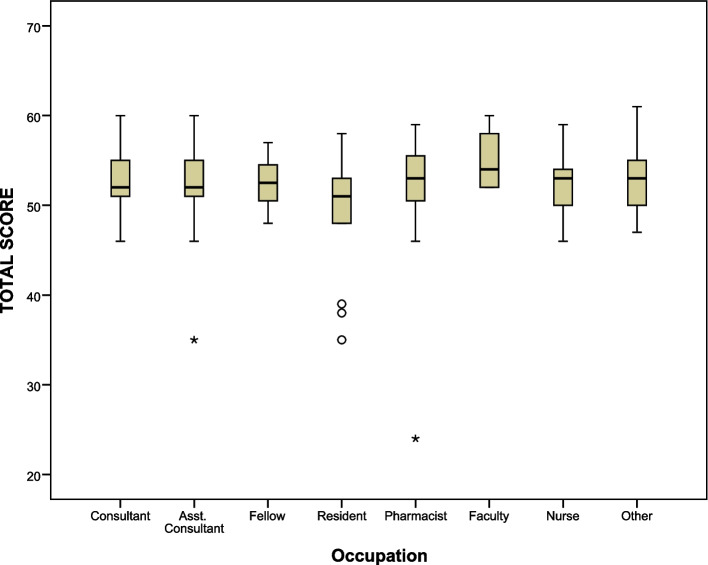
Box plot of total knowledge score by occupation

**Fig. 3:**
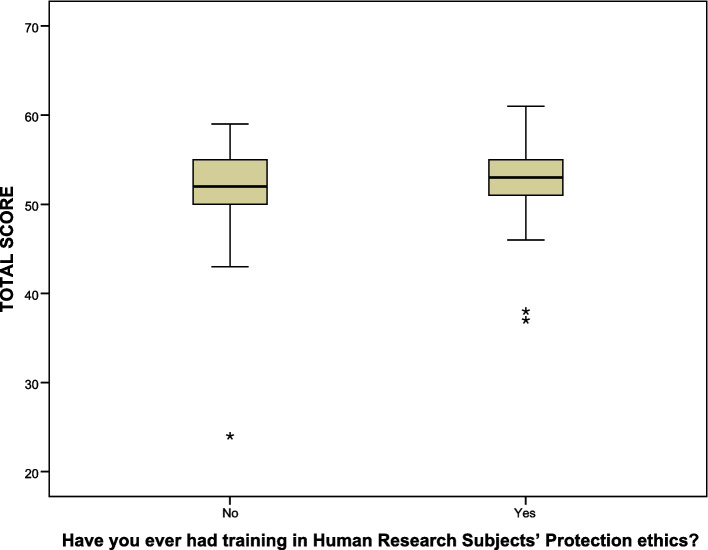
Box plot of the total knowledge score by prior training

The current study also compared the test performance across some demographic and research backgrounds. The findings showed strong statistical differences in the average total knowledge score by occupations (*p*-value = 0.017). However, post hoc analysis indicated that consultants (53.1 ± 3.1, *p*-value < 0.001), assistant consultants (52.7 ± 4.1, *p*-value = 0.001), fellows (52.0 ± 3.2, *p*-value = 0.024), faculties (55.2 ± 3.6, *p*-value = 0.004), pharmacists (52.3 ± 6.1, *p*-value = 0.008), nurses (52.4 ± 3.8, *p*-value = 0.003), and other occupations (51.7 ± 5.1, *p*-value = 0.025) scored significantly-higher than residents (48.8 ± 6.9) as shown in Fig. 2, while other pairwise comparisons showed no significant statistical differences, indicating similar knowledge levels (*p*-values > 0.05). Furthermore, participants with prior training on human subjects’ rights had significantly higher average knowledge scores (52.6 ± 3.9) than those without prior training (51.3 ± 5.7, *p*-value = 0.040) as shown in Fig. 3. Significant positive correlations were observed between total knowledge score and research experiences (*r* = 0.163, *p*-value = 0.039) and number of published articles (*r* = 0.203, *p*-value < 0001). No significant statistical differences in average knowledge scores were found by other demographic characteristics.

## Discussion

Based on a rigorous methodology for developing a valid and reliable questionnaire, especially in terms of face and content validity, the current study demonstrated the validity of the current instrument and its ability to achieve measurement objectives [29, 30]. I-CVIs and S-CVI/UA (universal agreement) were used to determine content validity. All parts of the questionnaire that scored higher than the benchmark found in the literature showed high content validity [23, 31].

Split-half reliability coefficient analysis of question items on researchers’ knowledge of human subjects’ rights as per scientific literature guidelines demonstrated good internal consistency reliability [32, 33]. This method is considered the best alternative to test–retest reliability if the retest is not applicable [33, 34]. As for researchers’ attitudes toward research ethics education, the Cronbach’s alpha showed good internal consistency and reliability for this scale [35, 36].

The results of item analysis on question items on knowledge showed an appropriate level of difficulty for most questions [27, 28]. Furthermore, most questions had appropriate levels of discrimination, either according to the guidelines proposed by Oosterhof [27] or a biserial correlation greater than 0.20 [28]. However, five out of 65 questions had unacceptable item difficulty and item discrimination (i.e., Q 2.2, Q 2.10, Q 2.15, Q 5.11, and Q 6.3). Three questions (2.2, 2.10, and 5.11) were retained to avoid random answers and reduce acquiescent and extreme response biases. Questions 2.2 and 2.10 were used to measure the basic aspects of informed consent elements [37]; Question 2.15 was considered one of the additional elements in informed consent as per CFR [18], and Question 5.11 was used to measure the knowledge of researchers’ responsibilities in handling adverse events [38]. Question 6.3 was removed because it had both a low I-CVI and a low discrimination index.

The current study also looked at intergroup differences in overall test knowledge and found that participants’ occupations and their prior training status in research ethics had a strong statistical impact. More specifically, consultants, assistant consultants, fellows, faculties, pharmacists, nurses, and others (e.g., research center members) delivered higher performance than residents. This indicates that residents are more likely to face a higher gap of knowledge about research ethics than others. Moreover, participants who received prior training in research ethics delivered better performance in the knowledge test than those who did not. Cummings et al. indicated that participants who had previously received ethics training performed better than participants who had not [39]. Therefore, the current instrument could be used to address knowledge gaps in research ethics. Notably, the overall performance on knowledge of all questionnaire question items revealed a knowledge gap in various aspects of clinical research ethics, which confirmed previous findings [15, 24, 39–41]. Furthermore, the current results of the study showed that most participants supported the need for education in clinical research ethics and preferred face-to-face learning modules to distance learning courses. Further education in clinical research ethics would improve investigators’ performance in ethics knowledge tests [42–45].

The questionnaire developed in this study is useful in the assessment of knowledge gaps among biomedical researchers on different aspects of clinical research ethics and their attitudes toward education on research ethics. Furthermore, it can be used to design educational intervention programs and test their acceptability, as certain topics are prioritized to address (or overcome) knowledge of deficits in clinical ethics. If the main goal of clinical research ethics is to facilitate investigators’ knowledge of and ethical actions regarding human subjects’ rights, these intervention programs must teach them the most important skills and knowledge of clinical research ethics and test them to see how well they know and use these skills [45].

Finally, though this study was successful in developing and validating a tool to assess researchers’ knowledge about human subjects’ rights and their attitudes toward education on research ethics education in the biomedical field using rigorous methods and a reasonable sample size, it had some potential limitations. First, we could not conduct a test–retest reliability analysis, even though split-half reliability showed good internal consistency of the knowledge items. Second, this study was conducted at KFMC and might not be representative and generalizable among other biomedical researchers on either local or international levels. Third, this study could not assess the tool for criterion validity owing to the lack of available validated tools and gold standards in the cited literature. Finally, this instrument only assessed the knowledge of clinical research ethics, not behavior, skills, or practice.

## Conclusion

This study has successfully developed a valid and reliable tool to assess researchers’ knowledge of human subjects’ rights and attitudes toward education on research ethics. The final version of the questionnaire included 64 question items on knowledge that covered contents in 6 main domains of research ethics and 10 items on attitudes toward research ethics education. This instrument could be useful in addressing gaps in the knowledge of human subjects’ rights and facilitating the development of educational intervention programs to set appropriate learning objectives. Other researchers are recommending continued efforts to use this novel tool to identify a robust questionnaire to measure the same construct. Finally, the methodology applied in this study can be utilized in developing similar assessment tools in clinical research ethics. This tool is valid to be used in European countries with minor changes.

### Supplementary Information

**Additional file 1.** First draft of the questionnaire.

**Additional file 2.** First revised questionnaire after face validity.

**Additional file 3.** Second revised questionnaire after content validity.

**Additional file 4.** Third revised questionnaire after pilot study.

**Additional file 5.** Final version of the questionnaire.

## Data Availability

The datasets used and/or analysed during the current study are available from the corresponding author on reasonable request.

## Abbreviations

CITI: Collaborative Institutional Training Initiative
CVI: The Content Validity Index
ECs: Ethics Committees
GCP: Good Clinical Practice
I-CVI: Item-level Content Validity Index
ICH-GCP: International Conference on Harmonization Good Clinical Practice
IQR: Interquartile Range
IRBs: Institutional Review Boards
KFMC: King Fahad Medical City
KSA: Kingdom of Saudi Arabia
NCBE: National Committee of Bioethics
NIDA: The National Institute on Drug Abuse
NIH: National Institutes of Health
REC: Research Ethics Committee
S-CVI: The Scale-level Content Validity Index
S-CVI/UA: Scale-level Content Validity Index, Universal Agreement calculation method
SD: Standard Deviation
