# Supplementary material for: Development and validation of a tool to assess researchers’ knowledge of human subjects’ rights and their attitudes toward research ethics education in Saudi Arabia
Source: BMC Med Ethics. 2023 Nov 2;24:94. doi: 10.1186/s12910-023-00968-z (PMC10623786; doi:10.1186/s12910-023-00968-z)
Supplement: Supplementary file 1 — Additional file 1. First draft of the questionnaire. [file 12910_2023_968_MOESM1_ESM.pdf]

Additional file 1

Development and validation of a tool to assess researchers' knowledge of human subjects' rights and their attitudes toward research ethics education in Saudi Arabia

First draft of the questionnaire

Part I. Demographic characteristics and professional characteristics

1. **Age** ☐☐ Years

2. **Gender** 1 ☐ M 2 ☐ F

3. **Nationality** 1 ☐ Saudi 2 ☐ non-Saudi

4. **Level of education**

   1 ☐ Diploma degree 2 ☐ Bachelor's degree

   3 ☐ Master's 4 ☐ Doctorate

   5 ☐ Subspecialty or Fellowship 6 ☐ Other, specify…………………

5. **Medical education graduates**

   1 ☐ Saudi Arabia 2 ☐ Arab countries

   3 ☐ Eastern Europe 4 ☐ Western Europe

   5 ☐ North America 6 ☐ Other, specify……………

6. **Occupation**

   1 ☐ Consultant 2 ☐ Asst. Consultant 3 ☐ Fellow

   4 ☐ Resident 5 ☐ Pharmacist 6 ☐ Faculty

   7 ☐ Nurse 8 ☐ Other, specify…………….

7. **Research experience** ☐☐ Years

8. **Number of research publications in medical journals** ☐☐☐

9. **Have you ever had training in Human Research Subjects' Protection ethics**?

   1 ☐ Yes 2 ☐ No 3 ☐ Not sure

10. If yes, please indicate which training you had: (you can choose more than one answer)

a. ☐ Good Clinical Practice (GCP) guidelines
b. ☐ National Institutes of Health (NIH) Research Ethics
c. ☐ Collaborative Institutional Training Initiative (CITI) Program
d. ☐ The Saudi National Committee of Bioethics (NCBE) certification
e. ☐ The National Institute on Drug Abuse (NIDA) Clinical Trials Network
f. ☐ Other, please specify …………...

11. Have you ever participated in research as a Principal Investigator (PI)?

1 ☐ Yes 2 ☐ No 3 ☐ Not sure

12. Have you ever participated in research as a Co-investigator (Co-PI)?

1 ☐ Yes 2 ☐ No 3 ☐ Not sure

13. How well do you know the "World Medical Association Declaration of Helsinki Ethical Principles for Medical Research Involving Human Subjects"?

1 ☐ Excellent knowledge
2 ☐ Very good knowledge
3 ☐ Average knowledge
4 ☐ Very little knowledge
5 ☐ I don't know what the Declartaion of Helsinki is

14. How well do you know the Implementing Regulations of the Law of Ethics of Research on Living Creatures issued by "The Saudi National Committee of Bioethics (NCBE)"?

1 ☐ Excellent knowledge
2 ☐ Very good knowledge
3 ☐ Average knowledge
4 ☐ Very little knowledge
5 ☐ I am not aware of the Saudi National Committee of Bioethics

15. Which of the following are considered ethical guidelines in research ethics?

1 ☐ Nuremberg Code,
2 ☐ Declaration of Helsinki,
3 ☐ Belmont Report,
4 ☐ Council of the International Organizations of the Medical Sciences (CIOMS)
5 ☐ All of the above

Part II. Knowledge of subjects' rights protection

1. **Knowledge of the basic and additional elements of informed consent:**

If you conduct clinical research on a human subject, which of the following elements should be part of the informed consent:

Items	Yes	No	I Don't Know
1. A statement that the study involves research	☐	☐	☐
2. An explanation of the purposes of the research is not a part of the informed consent	☐	☐	☐
3. An explanation of the expected duration of the subject's participation	☐	☐	☐
4. A description of the procedures to be followed, and identification of any procedures which are experimental	☐	☐	☐
5. A description of any reasonably foreseeable risks or discomforts to the Subject	☐	☐	☐
6. A description of any benefits to the subject or to others which may reasonably be expected from the research	☐	☐	☐
7. A non-disclosure of appropriate alternative procedures or courses of treatment, that might be advantageous to the subject	☐	☐	☐
8. A statement describing to what extent records will be kept confidentially, including a description of how many have access to research records	☐	☐	☐
9. An explanation as to whether any compensation and an explanation as to whether any medical treatments are available if injury occurs	☐	☐	☐
10. Primary investigators' contact information for answers to pertinent questions is not part of the informed consent	☐	☐	☐
11. A statement that participation is voluntary, that refusal to participate will involve no penalty or loss of benefits to which the subject is otherwise entitled, and that the subject may discontinue participation at any time without penalty or loss of benefits to which the subject is otherwise Entitled	☐	☐	☐
12. A statement that the particular treatment or procedure may involve risks to the subject (or to the embryo or fetus, if the subject is or may become pregnant) which are currently unforeseeable	☐	☐	☐
13. Anticipated circumstances under which the subject's participation may be terminated by the investigator without regard to the subject's consent	☐	☐	☐
14. Any additional costs to the subject that may result from participation in the research	☐	☐	☐
15. The consequences of a subject's decision to withdraw from the research and procedures for orderly termination of participation by the subject	☐	☐	☐

16. A statement that significant new findings developed during the course of the research which may relate to the subject's willingness to continue participation will be provided to the subject	☐	☐	☐
17. The approximate number of subjects involved in the study	☐	☐	☐

2. Knowledge about Institutional Review Board (IRB)/Research Ethics Committees (REC)

**The role of the IRB/REC is to:**	**Yes**	**No**	**I Don't Know**
1. Protect the rights, safety, and wellbeing of research subjects	☐	☐	☐
2. Ensure that Clinical research is conducted in study protocol, its amendments, and in accordance with the ethical principles, Declaration of Helsinki, GCP and the applicable regulatory requirement(s)	☐	☐	☐
3. Ensure that the reported data is accurate and credible	☐	☐	☐
4. Review the scientific design of the research	☐	☐	☐
5. Review and approve the informed consent whenever needed	☐	☐	☐
6. Review the ethical aspects of the research	☐	☐	☐
7. Monitor all approved research activities based on the anticipated risk only	☐	☐	☐
8. To observe study performance, examine the informed consent process, and audit the progress of any study under its jurisdiction	☐	☐	☐
9. To suspend, terminate, or impose restrictions on previously approved on-going research projects if protocol is violated	☐	☐	☐
10. Review All adverse events or adverse drug reactions that are both serious and unexpected only	☐	☐	☐

3. **Knowledge about safety reporting issues in clinical research**

Kindly indicate whether the below items related to safety reporting issues in clinical research are correct or not

**Items:**	**Yes**	**No**	**I Don't Know**
1. Adverse Drug Reaction defined as all noxious and unintended responses to a medicinal product related to any dose	☐	☐	☐
2. Adverse Event is any untoward medical occurrence in a patient or clinical investigation subject administered a pharmaceutical product and which does not necessarily have a causal relationship with this Treatment	☐	☐	☐
3. Serious adverse event or serious adverse drug reaction is any untoward medical occurrence that at any dose results in death, life-threatening, requires inpatient hospitalization or prolongation of existing hospitalization, results in persistent or significant disability/incapacity, or a congenital anomaly/birth defect	☐	☐	☐
4. All serious adverse event should be reported immediately to the sponsor and IRB within 24 hours from acknowledging the event	☐	☐	☐
5. All adverse events should be reported immediately to the sponsor and IRB within 24 hours from acknowledging the event	☐	☐	☐
6. It is the IRB responsibility to make an assessment of intensity, causality, expectedness and seriousness of adverse event/reactions	☐	☐	☐

4. **Knowledge of researchers' responsibilities in clinical research:**

**Items:**	**Yes**	**No**	**I Don't Know**
1. The researchers should be properly qualified to by education, training, and experience to assume responsibility for the conduct of the study	☐	☐	☐
2. The researchers should be thoroughly familiar with the investigational product (IP) and its appropriate use	☐	☐	☐
3. The researchers should have sufficient time to properly conduct and complete the study	☐	☐	☐
4. The researchers should have available adequate number of qualified staff and adequate facilities to to conduct the study properly and safely	☐	☐	☐
5. The researchers should ensure that all the research team are adequately informed about the protocol, the investigational product(s), and their study-related duties and functions	☐	☐	☐
6. The researchers should be able to recruit sufficient number of subjects within the enrollment timeline	☐	☐	☐

7. The researchers should provide adequate medical care for participants who experience adverse events	☐	☐	☐
8. The researchers should not notify the participant's primary care physician of his/her participation in a research study (as appropriate if authorized by subject/LAR)	☐	☐	☐
9. The researchers should submit IRB/IEC application and provide the IRB/IEC with all research documents before and throughout the study period	☐	☐	☐
10. The researchers should obtain written approval from the IRB before the study begins and prior to implementation of any subsequent changes to the protocol	☐	☐	☐
11. The researcher can deviate in the study protocol without written approval from the sponsor and prior review/approval from the IRB in a non-emergency situation	☐	☐	☐
12. The researcher can deviate in the study protocol to eliminate an immediate hazard to study subjects	☐	☐	☐
13. The researcher is not responsible for the Investigational Product, its usage, storage, and destruction	☐	☐	☐
14. The researcher should avoid coercion or undue influence on the research subjects	☐	☐	☐
15. The researcher should avoid language that causes the subject or the subject's legally acceptable representative to waive any legal rights	☐	☐	☐
16. The researcher is not responsible for documenting and reporting any adverse events and serious adverse events	☐	☐	☐
17. The researcher is responsible for ensuring that any protocol changes are not implemented prior to IRB/IEC review and receipt of IRB/IEC approval documentation	☐	☐	☐
18. The researcher is responsible for documenting and reporting any adverse events and serious adverse events	☐	☐	☐
19. The researcher is not responsible for all aspects of the study conduct	☐	☐	☐

5. Others: knowledge of researchers' subjects' rights protection

Items:	Yes	No	I Don't Know
1. The subject should have enough time to decide whether or not to be in the research study, and to make that decision without any pressure from the people who are conducting the research	☐	☐	☐
2. Informed consents must be obtained by the study investigator, or a person designated by investigator	☐	☐	☐
3. The subject's acceptable representative can not sign the informed consent on behalf of the research subject if he is mentally competent	☐	☐	☐
4. If a subject is illiterate, an impartial witness should be present during the entire informed consent discussion	☐	☐	☐
5. A written informed consent form and any other written information to be provided to subjects	☐	☐	☐
6. The subject or the subject's legally acceptable representative should be provided with an ample time to inquire about details of the research and to decide whether or not to participate in the research	☐	☐	☐
7. The subject should not be able to know how much money the investigator is making from the clinical study	☐	☐	☐
8. The written informed consent form should be signed and personally dated by the subject or by the subject's legally acceptable representative, and by the person who conducted the informed consent discussion	☐	☐	☐
9. In emergency clinical trials, when prior consent of the subject or the subject's legally acceptable representative is not possible, the informed consent can be waived	☐	☐	☐
10. An assent should be taken from the child as well as a written informed consent from the child's parent/ guardian	☐	☐	☐

6. Clinical research scenario:

1. Ms. Mary comes to the clinic to have her blood draw for routine laboratories. The investigator takes a little more than usual for research purpose. Which of the following is true?

1 ☐ The investigator does not have to tell Mary the purpose of taking more blood.
2 ☐ The investigator does not have to tell Mary whether the blood will be used in research.
3 ☐ The investigator should have asked for permission and the informed consent from Mary
4 ☐ It is expected that patients participate in research without patients' knowledge

2. If Ms. Mary expects that the blood will be used in research, she can do which of the following:

1 ☐ Ask the investigator to withdraw this sample
2 ☐ Complain to the hospital director
3 ☐ Tell her family members, colleagues, and friends not to go to this doctor

4 ☐ Tell the media in order to warn the public from dealing with this doctor and this clinic
5 ☐ Do nothing as nothing will be done about it

3. If eighty patients from the outpatient clinic were enrolled in research. The aim of the research was to differentiate between two different treatment modalities in the management of periodontal in-trousseaus bony defects which of the following must be done to ensure the confidentiality (you can cross one answer only)

1 ☐ Patients' research files should be coded to ensure patients' confidentiality.
2 ☐ No need for confidentiality as the procedures are common in dental practice.
3 ☐ It is left to the investigator to decide whether to keep the research data confidential or not.
4 ☐ The dean or head of the department is the one to decide regarding the provisions of confidentiality.

Part III. Researchers' attitudes toward research ethics education

Kindly indicate your opinion about the below items related to research ethics education

**Items:**	**Strongly agree**	**Agree**	**Neutral**	**Disagree**	**Strongly disagree**
**1.** Research ethics should be taught as a mandatory undergraduate module	☐	☐	☐	☐	☐
2. Research ethics should be taught as a mandatory postgraduate module	☐	☐	☐	☐	☐
3. All investigators should have some training in research ethics	☐	☐	☐	☐	☐
4. I prefer face-to-face as the most effective research ethics teaching methods	☐	☐	☐	☐	☐
5. I prefer distance-learning as the most effective research ethics teaching methods	☐	☐	☐	☐	☐
6. I prefer hands-on and case scenarios during teaching method	☐	☐	☐	☐	☐
7. I prefer research ethics post education exam to assess my knowledge	☐	☐	☐	☐	☐
8. I prefer reading course material in advance of the ethics training course	☐	☐	☐	☐	☐
9. Research ethics education should be mandatory for health care professionals	☐	☐	☐	☐	☐
10. The IRB members should be educated in research ethics	☐	☐	☐	☐	☐
